# 6,6′-Dimeth­oxy-2,2′-[4,5-dimethyl-*o*-phenyl­enebis(nitrilo­methyl­idyne)]diphenol monohydrate

**DOI:** 10.1107/S1600536810002916

**Published:** 2010-02-06

**Authors:** Hadi Kargar, Reza Kia, Islam Ullah Khan, Atefeh Sahraei

**Affiliations:** a Department of Chemistry, School of Science, Payame Noor University (PNU), Ardakan, Yazd, Iran; b Department of Chemistry, Science and Research Campus, Islamic Azad University, Poonak, Tehran, Iran; c Materials Chemistry Laboratory, Department of Chemistry, GC University, Lahore 54000, Pakistan

## Abstract

In the title compound, C_24_H_24_N_2_O_4_·H_2_O, the dihedral angles between the central benzene ring and the two outer benzene rings of the Schiff base are 65.06 (9) and 3.02 (9)°. Strong intra­molecular O—H⋯N hydrogen bonds generate *S*(6) ring motifs. The H atoms of the water mol­ecule act as donors in the formation of bifurcated O—H⋯(O,O) inter­molecular hydrogen bonds with the O atoms of the hydr­oxy and meth­oxy groups with *R*_1_^2^(5) ring motifs; these may influence the mol­ecular conformation.

## Related literature

For bond-length data, see: Allen *et al.* (1987 ▶). For hydrogen-bond motifs, see: Bernstein *et al.* (1995 ▶). For related structures, see: Cakir *et al.* (2002 ▶); Eltayeb & Ahmed (2005 ▶); Eltayeb *et al.* (2007 ▶); Kargar *et al.* (2009 ▶). For background to the applications of Schiff base ligands as thermochromic and photochromic materials, see: Hajioudis *et al.* (1987 ▶).
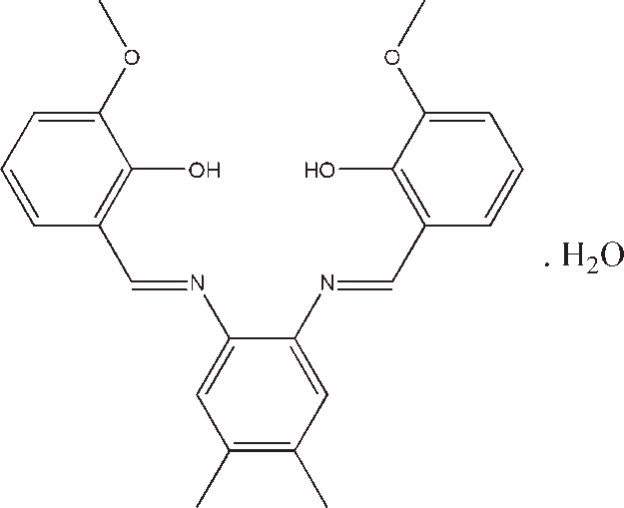


## Experimental

### 

#### Crystal data

C_24_H_24_N_2_O_4_·H_2_O
*M* *_r_* = 422.47
Triclinic, 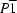

*a* = 8.7431 (5) Å
*b* = 10.3049 (6) Å
*c* = 13.6614 (7) Å
α = 69.556 (3)°
β = 83.846 (3)°
γ = 70.280 (3)°
*V* = 1085.6 (1) Å^3^
*Z* = 2
Mo *K*α radiation
μ = 0.09 mm^−1^
*T* = 296 K
0.30 × 0.20 × 0.15 mm

#### Data collection

Bruker SMART APEXII CCD area-detector diffractometer
Absorption correction: multi-scan (*SADABS*; Bruker, 2005 ▶) *T*_min_ = 0.973, *T*_max_ = 0.987
23270 measured reflections
5369 independent reflections
2912 reflections with *I* > 2*I*)
*R*_int_ = 0.031

#### Refinement

*R*[*F* ^2^ > 2σ(*F* ^2^)] = 0.046
*wR*(*F* ^2^) = 0.141
*S* = 1.01
5369 reflections
284 parameters
H-atom parameters constrained
Δρ_max_ = 0.20 e Å^−3^
Δρ_min_ = −0.18 e Å^−3^

### 

Data collection: *APEX2* (Bruker, 2005 ▶); cell refinement: *SAINT* (Bruker, 2005 ▶); data reduction: *SAINT*; program(s) used to solve structure: *SHELXTL* (Sheldrick, 2008 ▶); program(s) used to refine structure: *SHELXTL*; molecular graphics: *SHELXTL*; software used to prepare material for publication: *SHELXTL* and *PLATON* (Spek, 2009 ▶).

## Supplementary Material

Crystal structure: contains datablocks global, I. DOI: 10.1107/S1600536810002916/lh2982sup1.cif

Structure factors: contains datablocks I. DOI: 10.1107/S1600536810002916/lh2982Isup2.hkl

Additional supplementary materials:  crystallographic information; 3D view; checkCIF report

## Figures and Tables

**Table 1:** Hydrogen-bond geometry (Å, °)

*D*—H⋯*A*	*D*—H	H⋯*A*	*D*⋯*A*	*D*—H⋯*A*
O1—H1⋯N1	0.96	1.72	2.5929 (18)	150
O2—H2⋯N2	0.96	1.66	2.5704 (18)	156
O1*W*—H1*W*⋯O1	0.97	2.21	3.050 (2)	144
O1*W*—H1*W*⋯O3	0.97	2.50	3.366 (2)	148
O1*W*—H2*W*⋯O2	0.97	2.15	3.079 (2)	160
O1*W*—H2*W*⋯O4	0.97	2.55	3.271 (2)	131

**Fig. 1.**
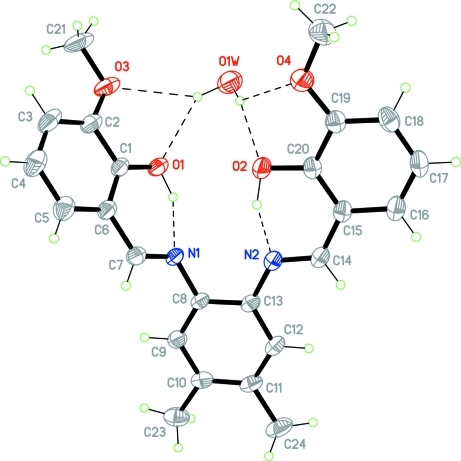
The asymmetric unit of the title compound, showing 30% probability displacement ellipsoids and the atomic numbering. Intra- and intermolecular hydrogen bonds are drawn as dashed lines.

**Fig. 2.**
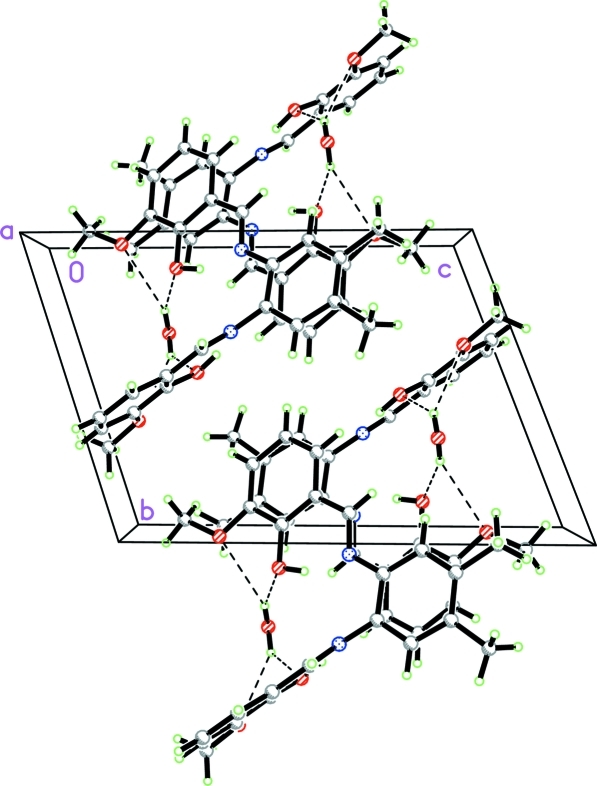
The crystal packing of the title compound wieved along the a-axis. Hydrogen bonds are shown as dashed lines.

## supplementary crystallographic information

### Comment

Schiff base ligands are one of the most prevalent systems in coordination chemistry. They can show thermochromic and photochromic properties (Hajioudis *et al.* (1987). As part of a general study of tetradenate Schiff bases (Kargar *et al.* 2009), we have determined the crystal structure of the title compound.

The asymmetric unit of the title compound, Fig. 1, comprises a Schiff base ligand and a water molecule of crystallization. The bond lengths (Allen *et al.,* 1987) and angles are within the normal ranges and comparable to previously reported structures (Eltayeb & Ahmed, 2005; Eltayeb *et al.*, 2007; Cakir *et al.* 2002; Kargar *et al.,* 2009 ). The dihedral angles between the central benzene ring and the two outer benzene rings of the Schiff base are 65.06 (9) and 3.02 (9)°. Strong intramolecular O—H···N hydrogen bonds generate *S(6)* ring motifs (Bernstein *et al.,* 1995). The hydrogen atoms of the water molecule form bifurcated intermolecular hydrogen bonds with the oxygen atoms of the hydroxy and methoxy groups with *R^2^_1_(5)* ring motifs (Bernstein *et al.,* 1995), which may, in part, influence the molecular configuration (Table 1). A view of part of the crystal structure is shown in Fig .2.

### Experimental

The title compound was synthesized by adding 3-methoxy-salicylaldehyde (4 mmol) to a solution of 4,5-dimethyl-*o*-phenylenediamine (2 mmol) in ethanol (20 ml). The mixture was refluxed with stirring for half an hour. The resultant yellow solution was filtered. Yellow single crystals of the title compound suitable for *X*-ray structure determination were recrystallized from ethanol by slow evaporation of the solvents at room temperature over several days.

### Refinement

H atoms of the hydroxy groups of the Schiff base and water were located in a difference Fourier map. Initially the O-H distances were restrained to 0.96 (1) and 0.98 (1) Å, respectively and in the final cycles of refinement these H atoms were allowed to ride on the parent O atom with U_iso_(H) = 1.5 U_eq_(O), see Table 1. The remaining H atoms were positioned geometrically with C-H = 0.93-0.96 Å and included in a riding model approximation with U_iso_ (H) = 1.2 or 1.5 U_eq_ (C). A rotating group model was used for the methyl groups.

### Crystal data

**Table:**

C_24_H_24_N_2_O_4_·H_2_O	*Z* = 2
*M**_r_* = 422.47	*F*(000) = 448
Triclinic, *P*1	*D*_x_ = 1.292 Mg m^−^^3^
Hall symbol: -P 1	Mo *K*α radiation, λ = 0.71073 Å
*a* = 8.7431 (5) Å	Cell parameters from 5124 reflections
*b* = 10.3049 (6) Å	θ = 2.2–25.0°
*c* = 13.6614 (7) Å	µ = 0.09 mm^−^^1^
α = 69.556 (3)°	*T* = 296 K
β = 83.846 (3)°	Block, yellow
γ = 70.280 (3)°	0.30 × 0.20 × 0.15 mm
*V* = 1085.6 (1) Å^3^	

### Data collection

**Table:**

Bruker SMART APEXII CCD area-detector diffractometer	5369 independent reflections
Radiation source: fine-focus sealed tube	2912 reflections with *I* > 2˘*I*)
graphite	*R*_int_ = 0.031
φ and ω scans	θ_max_ = 28.3°, θ_min_ = 2.2°
Absorption correction: multi-scan (*SADABS*; Bruker, 2005)	*h* = −11→11
*T*_min_ = 0.973, *T*_max_ = 0.987	*k* = −12→13
23270 measured reflections	*l* = −18→18

### Refinement

**Table:**

Refinement on *F*^2^	Primary atom site location: structure-invariant direct methods
Least-squares matrix: full	Secondary atom site location: difference Fourier map
*R*[*F*^2^ > 2σ(*F*^2^)] = 0.046	Hydrogen site location: inferred from neighbouring sites
*wR*(*F*^2^) = 0.141	H-atom parameters constrained
*S* = 1.00	*w* = 1/[σ^2^(*F*_o_^2^) + (0.0622*P*)^2^ + 0.1326*P*] where *P* = (*F*_o_^2^ + 2*F*_c_^2^)/3
5369 reflections	(Δ/σ)_max_ < 0.001
284 parameters	Δρ_max_ = 0.20 e Å^−^^3^
0 restraints	Δρ_min_ = −0.18 e Å^−^^3^

### Special details

**Table:**

Geometry. All esds (except the esd in the dihedral angle between two l.s. planes) are estimated using the full covariance matrix. The cell esds are taken into account individually in the estimation of esds in distances, angles and torsion angles; correlations between esds in cell parameters are only used when they are defined by crystal symmetry. An approximate (isotropic) treatment of cell esds is used for estimating esds involving l.s. planes.
Refinement. Refinement of F^2^ against ALL reflections. The weighted R-factor wR and goodness of fit S are based on F^2^, conventional R-factors R are based on F, with F set to zero for negative F^2^. The threshold expression of F^2^ > 2sigma(F^2^) is used only for calculating R-factors(gt) etc. and is not relevant to the choice of reflections for refinement. R-factors based on F^2^ are statistically about twice as large as those based on F, and R- factors based on ALL data will be even larger.

### Fractional atomic coordinates and isotropic or equivalent isotropic displacement parameters (Å^2^)

**Table:**

	*x*	*y*	*z*	*U*_iso_*/*U*_eq_	
O1	0.48196 (13)	0.46281 (13)	0.27727 (9)	0.0574 (3)	
H1	0.5531	0.4027	0.3362	0.086*	
O2	0.58907 (14)	0.10375 (12)	0.31399 (9)	0.0563 (3)	
H2	0.6463	0.1102	0.3678	0.084*	
O3	0.34071 (17)	0.62867 (15)	0.09838 (10)	0.0749 (4)	
O4	0.45471 (16)	0.01221 (14)	0.20011 (10)	0.0692 (4)	
N1	0.74540 (15)	0.32782 (13)	0.39027 (10)	0.0436 (3)	
N2	0.74691 (15)	0.04912 (14)	0.47979 (10)	0.0438 (3)	
C1	0.5789 (2)	0.51020 (17)	0.19880 (12)	0.0465 (4)	
C2	0.5055 (2)	0.59934 (18)	0.10105 (13)	0.0549 (5)	
C3	0.5987 (3)	0.6496 (2)	0.01908 (14)	0.0687 (6)	
H3A	0.5502	0.7081	−0.0459	0.082*	
C4	0.7642 (3)	0.6143 (2)	0.03171 (15)	0.0733 (6)	
H4A	0.8262	0.6484	−0.0249	0.088*	
C5	0.8378 (2)	0.5294 (2)	0.12719 (14)	0.0630 (5)	
H5A	0.9489	0.5076	0.1354	0.076*	
C6	0.7458 (2)	0.47583 (17)	0.21197 (12)	0.0469 (4)	
C7	0.8237 (2)	0.38539 (17)	0.31301 (13)	0.0466 (4)	
H7A	0.9335	0.3692	0.3214	0.056*	
C8	0.82177 (17)	0.24595 (16)	0.48957 (11)	0.0401 (4)	
C9	0.88484 (19)	0.30867 (18)	0.54329 (13)	0.0475 (4)	
H9A	0.8844	0.4046	0.5110	0.057*	
C10	0.94858 (19)	0.2329 (2)	0.64369 (13)	0.0496 (4)	
C11	0.95310 (19)	0.0878 (2)	0.69018 (12)	0.0507 (4)	
C12	0.88856 (19)	0.02546 (18)	0.63693 (12)	0.0493 (4)	
H12A	0.8914	−0.0712	0.6685	0.059*	
C13	0.81949 (17)	0.10333 (17)	0.53748 (12)	0.0410 (4)	
C14	0.74023 (18)	−0.08139 (17)	0.51154 (13)	0.0467 (4)	
H14A	0.7864	−0.1459	0.5759	0.056*	
C15	0.66348 (18)	−0.13050 (17)	0.45037 (13)	0.0455 (4)	
C16	0.6581 (2)	−0.27411 (18)	0.48757 (15)	0.0563 (5)	
H16A	0.7039	−0.3370	0.5524	0.068*	
C17	0.5870 (2)	−0.32238 (19)	0.43002 (16)	0.0605 (5)	
H17A	0.5842	−0.4179	0.4556	0.073*	
C18	0.5181 (2)	−0.2296 (2)	0.33301 (16)	0.0589 (5)	
H18A	0.4699	−0.2637	0.2940	0.071*	
C19	0.5206 (2)	−0.08833 (19)	0.29418 (14)	0.0517 (4)	
C20	0.59181 (18)	−0.03598 (17)	0.35303 (13)	0.0458 (4)	
C21	0.2574 (3)	0.7227 (2)	0.00270 (16)	0.0918 (8)	
H21A	0.1438	0.7335	0.0106	0.138*	
H21B	0.3006	0.6814	−0.0514	0.138*	
H21C	0.2715	0.8168	−0.0157	0.138*	
C22	0.4007 (3)	−0.0392 (3)	0.13155 (17)	0.0840 (7)	
H22A	0.3546	0.0412	0.0697	0.126*	
H22B	0.3198	−0.0834	0.1654	0.126*	
H22C	0.4908	−0.1104	0.1129	0.126*	
C23	1.0072 (2)	0.3092 (2)	0.70012 (15)	0.0693 (6)	
H23A	0.9946	0.4077	0.6554	0.104*	
H23B	1.1198	0.2580	0.7188	0.104*	
H23C	0.9447	0.3106	0.7622	0.104*	
C24	1.0284 (2)	−0.0036 (3)	0.79731 (14)	0.0789 (7)	
H24A	0.9768	0.0447	0.8466	0.118*	
H24B	1.1423	−0.0157	0.7950	0.118*	
H24C	1.0137	−0.0977	0.8183	0.118*	
O1W	0.25045 (16)	0.32247 (16)	0.23580 (10)	0.0795 (4)	
H1W	0.2953	0.4012	0.2238	0.119*	
H2W	0.3547	0.2463	0.2467	0.119*	

### Atomic displacement parameters (Å^2^)

**Table:**

	*U*^11^	*U*^22^	*U*^33^	*U*^12^	*U*^13^	*U*^23^
O1	0.0557 (7)	0.0600 (8)	0.0444 (7)	−0.0177 (6)	−0.0123 (6)	−0.0001 (6)
O2	0.0718 (8)	0.0398 (7)	0.0541 (7)	−0.0176 (6)	−0.0099 (6)	−0.0094 (5)
O3	0.0744 (9)	0.0733 (9)	0.0556 (8)	−0.0061 (7)	−0.0298 (7)	−0.0043 (7)
O4	0.0853 (9)	0.0595 (8)	0.0662 (9)	−0.0225 (7)	−0.0143 (7)	−0.0214 (7)
N1	0.0496 (8)	0.0390 (7)	0.0354 (7)	−0.0115 (6)	−0.0089 (6)	−0.0044 (6)
N2	0.0436 (7)	0.0394 (8)	0.0416 (8)	−0.0102 (6)	0.0013 (6)	−0.0088 (6)
C1	0.0625 (10)	0.0373 (9)	0.0362 (9)	−0.0140 (8)	−0.0068 (8)	−0.0078 (7)
C2	0.0712 (12)	0.0433 (10)	0.0424 (10)	−0.0082 (9)	−0.0148 (9)	−0.0104 (8)
C3	0.1010 (17)	0.0537 (12)	0.0371 (10)	−0.0130 (11)	−0.0127 (10)	−0.0058 (9)
C4	0.0947 (17)	0.0735 (14)	0.0409 (11)	−0.0281 (12)	0.0079 (10)	−0.0076 (10)
C5	0.0733 (12)	0.0650 (12)	0.0466 (11)	−0.0248 (10)	0.0049 (9)	−0.0125 (9)
C6	0.0592 (10)	0.0412 (9)	0.0377 (9)	−0.0143 (8)	−0.0039 (8)	−0.0108 (7)
C7	0.0499 (9)	0.0434 (9)	0.0439 (10)	−0.0111 (8)	−0.0065 (8)	−0.0130 (8)
C8	0.0354 (8)	0.0404 (9)	0.0347 (8)	−0.0052 (6)	−0.0040 (6)	−0.0064 (7)
C9	0.0458 (9)	0.0467 (10)	0.0458 (10)	−0.0111 (7)	−0.0050 (7)	−0.0125 (8)
C10	0.0394 (9)	0.0627 (11)	0.0426 (9)	−0.0089 (8)	−0.0027 (7)	−0.0192 (9)
C11	0.0405 (9)	0.0648 (12)	0.0348 (9)	−0.0055 (8)	−0.0033 (7)	−0.0123 (8)
C12	0.0457 (9)	0.0437 (9)	0.0400 (9)	−0.0032 (7)	−0.0007 (7)	−0.0026 (7)
C13	0.0352 (8)	0.0433 (9)	0.0363 (8)	−0.0057 (7)	−0.0001 (6)	−0.0099 (7)
C14	0.0420 (9)	0.0428 (10)	0.0437 (9)	−0.0084 (7)	0.0034 (7)	−0.0064 (8)
C15	0.0383 (8)	0.0391 (9)	0.0527 (10)	−0.0088 (7)	0.0081 (7)	−0.0132 (8)
C16	0.0506 (10)	0.0421 (10)	0.0660 (12)	−0.0133 (8)	0.0086 (9)	−0.0099 (9)
C17	0.0549 (11)	0.0407 (10)	0.0834 (14)	−0.0200 (8)	0.0131 (10)	−0.0169 (10)
C18	0.0511 (10)	0.0542 (11)	0.0801 (14)	−0.0199 (9)	0.0106 (10)	−0.0329 (11)
C19	0.0472 (9)	0.0464 (10)	0.0590 (11)	−0.0115 (8)	0.0041 (8)	−0.0190 (9)
C20	0.0415 (9)	0.0387 (9)	0.0554 (10)	−0.0116 (7)	0.0079 (8)	−0.0171 (8)
C21	0.1047 (17)	0.0766 (15)	0.0687 (14)	0.0037 (13)	−0.0514 (13)	−0.0104 (12)
C22	0.1109 (18)	0.0963 (17)	0.0642 (14)	−0.0490 (14)	0.0015 (12)	−0.0350 (13)
C23	0.0644 (12)	0.0936 (16)	0.0568 (12)	−0.0233 (11)	−0.0064 (9)	−0.0333 (11)
C24	0.0728 (13)	0.0990 (17)	0.0419 (11)	−0.0162 (12)	−0.0161 (9)	−0.0021 (11)
O1W	0.0693 (9)	0.0887 (11)	0.0677 (9)	−0.0254 (8)	−0.0075 (7)	−0.0086 (8)

### Geometric parameters (Å, °)

**Table:**

O1—C1	1.3511 (19)	C11—C12	1.385 (2)
O1—H1	0.9574	C11—C24	1.512 (2)
O2—C20	1.3422 (18)	C12—C13	1.394 (2)
O2—H2	0.9642	C12—H12A	0.9300
O3—C2	1.371 (2)	C14—C15	1.432 (2)
O3—C21	1.422 (2)	C14—H14A	0.9300
O4—C19	1.372 (2)	C15—C16	1.402 (2)
O4—C22	1.411 (2)	C15—C20	1.404 (2)
N1—C7	1.271 (2)	C16—C17	1.356 (3)
N1—C8	1.4186 (18)	C16—H16A	0.9300
N2—C14	1.281 (2)	C17—C18	1.389 (3)
N2—C13	1.411 (2)	C17—H17A	0.9300
C1—C6	1.396 (2)	C18—C19	1.372 (2)
C1—C2	1.403 (2)	C18—H18A	0.9300
C2—C3	1.367 (3)	C19—C20	1.401 (2)
C3—C4	1.382 (3)	C21—H21A	0.9600
C3—H3A	0.9300	C21—H21B	0.9600
C4—C5	1.373 (3)	C21—H21C	0.9600
C4—H4A	0.9300	C22—H22A	0.9600
C5—C6	1.395 (2)	C22—H22B	0.9600
C5—H5A	0.9300	C22—H22C	0.9600
C6—C7	1.453 (2)	C23—H23A	0.9600
C7—H7A	0.9300	C23—H23B	0.9600
C8—C9	1.385 (2)	C23—H23C	0.9600
C8—C13	1.391 (2)	C24—H24A	0.9600
C9—C10	1.387 (2)	C24—H24B	0.9600
C9—H9A	0.9300	C24—H24C	0.9600
C10—C11	1.394 (2)	O1W—H1W	0.9731
C10—C23	1.502 (2)	O1W—H2W	0.9664
			
C1—O1—H1	105.3	N2—C14—H14A	119.2
C20—O2—H2	101.6	C15—C14—H14A	119.2
C2—O3—C21	117.48 (16)	C16—C15—C20	119.09 (16)
C19—O4—C22	117.25 (15)	C16—C15—C14	120.16 (16)
C7—N1—C8	120.70 (13)	C20—C15—C14	120.75 (15)
C14—N2—C13	123.85 (14)	C17—C16—C15	120.75 (17)
O1—C1—C6	122.82 (14)	C17—C16—H16A	119.6
O1—C1—C2	117.44 (16)	C15—C16—H16A	119.6
C6—C1—C2	119.74 (16)	C16—C17—C18	120.21 (17)
C3—C2—O3	125.95 (16)	C16—C17—H17A	119.9
C3—C2—C1	119.61 (18)	C18—C17—H17A	119.9
O3—C2—C1	114.44 (16)	C19—C18—C17	120.71 (18)
C2—C3—C4	120.71 (17)	C19—C18—H18A	119.6
C2—C3—H3A	119.6	C17—C18—H18A	119.6
C4—C3—H3A	119.6	C18—C19—O4	125.26 (17)
C5—C4—C3	120.52 (19)	C18—C19—C20	119.90 (17)
C5—C4—H4A	119.7	O4—C19—C20	114.83 (15)
C3—C4—H4A	119.7	O2—C20—C19	118.51 (15)
C4—C5—C6	119.97 (19)	O2—C20—C15	122.17 (15)
C4—C5—H5A	120.0	C19—C20—C15	119.32 (15)
C6—C5—H5A	120.0	O3—C21—H21A	109.5
C5—C6—C1	119.44 (15)	O3—C21—H21B	109.5
C5—C6—C7	120.03 (16)	H21A—C21—H21B	109.5
C1—C6—C7	120.54 (15)	O3—C21—H21C	109.5
N1—C7—C6	121.65 (15)	H21A—C21—H21C	109.5
N1—C7—H7A	119.2	H21B—C21—H21C	109.5
C6—C7—H7A	119.2	O4—C22—H22A	109.5
C9—C8—C13	119.56 (14)	O4—C22—H22B	109.5
C9—C8—N1	121.60 (14)	H22A—C22—H22B	109.5
C13—C8—N1	118.58 (14)	O4—C22—H22C	109.5
C8—C9—C10	122.06 (16)	H22A—C22—H22C	109.5
C8—C9—H9A	119.0	H22B—C22—H22C	109.5
C10—C9—H9A	119.0	C10—C23—H23A	109.5
C9—C10—C11	118.54 (16)	C10—C23—H23B	109.5
C9—C10—C23	119.63 (17)	H23A—C23—H23B	109.5
C11—C10—C23	121.82 (15)	C10—C23—H23C	109.5
C12—C11—C10	119.41 (14)	H23A—C23—H23C	109.5
C12—C11—C24	119.24 (17)	H23B—C23—H23C	109.5
C10—C11—C24	121.35 (17)	C11—C24—H24A	109.5
C11—C12—C13	121.97 (16)	C11—C24—H24B	109.5
C11—C12—H12A	119.0	H24A—C24—H24B	109.5
C13—C12—H12A	119.0	C11—C24—H24C	109.5
C8—C13—C12	118.37 (15)	H24A—C24—H24C	109.5
C8—C13—N2	116.34 (13)	H24B—C24—H24C	109.5
C12—C13—N2	125.28 (15)	H1W—O1W—H2W	95.0
N2—C14—C15	121.63 (15)		
			
C21—O3—C2—C3	−1.7 (3)	C10—C11—C12—C13	0.3 (2)
C21—O3—C2—C1	177.79 (17)	C24—C11—C12—C13	−179.15 (15)
O1—C1—C2—C3	−179.75 (16)	C9—C8—C13—C12	−3.1 (2)
C6—C1—C2—C3	1.2 (3)	N1—C8—C13—C12	−177.31 (14)
O1—C1—C2—O3	0.7 (2)	C9—C8—C13—N2	177.27 (13)
C6—C1—C2—O3	−178.33 (15)	N1—C8—C13—N2	3.0 (2)
O3—C2—C3—C4	178.97 (18)	C11—C12—C13—C8	2.5 (2)
C1—C2—C3—C4	−0.5 (3)	C11—C12—C13—N2	−177.89 (14)
C2—C3—C4—C5	−0.7 (3)	C14—N2—C13—C8	177.54 (14)
C3—C4—C5—C6	1.1 (3)	C14—N2—C13—C12	−2.1 (2)
C4—C5—C6—C1	−0.4 (3)	C13—N2—C14—C15	179.30 (14)
C4—C5—C6—C7	179.75 (18)	N2—C14—C15—C16	−179.90 (15)
O1—C1—C6—C5	−179.76 (16)	N2—C14—C15—C20	−0.3 (2)
C2—C1—C6—C5	−0.7 (2)	C20—C15—C16—C17	1.0 (2)
O1—C1—C6—C7	0.1 (2)	C14—C15—C16—C17	−179.45 (16)
C2—C1—C6—C7	179.11 (15)	C15—C16—C17—C18	0.0 (3)
C8—N1—C7—C6	−176.84 (14)	C16—C17—C18—C19	−0.2 (3)
C5—C6—C7—N1	−175.85 (16)	C17—C18—C19—O4	−179.23 (16)
C1—C6—C7—N1	4.3 (2)	C17—C18—C19—C20	−0.6 (3)
C7—N1—C8—C9	61.2 (2)	C22—O4—C19—C18	−9.5 (3)
C7—N1—C8—C13	−124.72 (17)	C22—O4—C19—C20	171.84 (16)
C13—C8—C9—C10	0.9 (2)	C18—C19—C20—O2	−178.50 (15)
N1—C8—C9—C10	174.99 (14)	O4—C19—C20—O2	0.3 (2)
C8—C9—C10—C11	1.9 (2)	C18—C19—C20—C15	1.5 (2)
C8—C9—C10—C23	−176.79 (15)	O4—C19—C20—C15	−179.69 (14)
C9—C10—C11—C12	−2.5 (2)	C16—C15—C20—O2	178.33 (14)
C23—C10—C11—C12	176.17 (15)	C14—C15—C20—O2	−1.3 (2)
C9—C10—C11—C24	176.97 (16)	C16—C15—C20—C19	−1.7 (2)
C23—C10—C11—C24	−4.4 (3)	C14—C15—C20—C19	178.70 (15)

### Hydrogen-bond geometry (Å, °)

**Table:**

*D*—H···*A*	*D*—H	H···*A*	*D*···*A*	*D*—H···*A*
O1—H1···N1	0.96	1.72	2.5929 (18)	150
O1W—H1W···O1	0.97	2.21	3.050 (2)	144
O1W—H1W···O3	0.97	2.50	3.366 (2)	148
O2—H2···N2	0.96	1.66	2.5704 (18)	156
O1W—H2W···O2	0.97	2.15	3.079 (2)	160
O1W—H2W···O4	0.97	2.55	3.271 (2)	131
